# Prevalence of cancer in the elderly: discrepancies between self-reported and registry data.

**DOI:** 10.1038/bjc.1997.74

**Published:** 1997

**Authors:** F. Berthier, P. Grosclaude, H. Bocquet, B. Faliu, F. Cayla, M. Machelard-Roumagnac

**Affiliations:** Laboratoire d'Epidémiologie et de Sante Publique, Faculté de Médecine,Toulouse, France.

## Abstract

Data on self-reported cancer by a sample of 3349 elderly persons in the south-west of France were validated against registry data in the same region: only 21% of the persons on the cancer registry reported occurrence of cancer. Breast cancer was found to be most frequently accurately reported.


British Journal of Cancer (1997) 75(3), 445–447
© 1997 Cancer Research Campaign

### Prevalence of cancer in the elderly: discrepancies between self-reported and registry data

**F Berthier[1], P Grosclaude[2], H Bocquet[1], B Faliu[2], F Cayla[3] and M Machelard-Roumagnac[4]**

[1]Laboratoire d'Épidémiologie et de Santé Publique, Faculté de Médecine, 37, Allées Jules Guesde, 31073 Toulouse Cedex; [2]Registre des Cancers du Tarn, Chemin des 3 Tarn, 81000 Albi; [3]Observatoire Régional de la Santé Midi-Pyrénées, Faculté de Médecine, 37, Allées Jules Guesde, 31073 Toulouse Cedex; [4]Centre de Lutte Contre le Cancer Claudius Regaud, 20–24 Rue du Pont Saint-Pierre, 31052 Toulouse Cedex, France

**Summary** Data on self-reported cancer by a sample of 3349 elderly persons in the south-west of France were validated against registry data in the same region: only 21% of the persons on the cancer registry reported occurrence of cancer. Breast cancer was found to be most frequently accurately reported.

**Keywords:** elderly; validity; questionnaire; registry records

In both large cohort and genetic studies, the absence of a population based cancer register for convenient identification of individuals with cancer often means that self-reported data are used. In such cases, only positive responses tend to be verified. However, relatively little attention has been focused on the real possibility of significant numbers of false-negative responses. In view of the high incidence of cancer in the elderly (Yancik and Ries, 1994; Coleman and Lutz, 1996), an increasing number of elderly people are likely to be included in future epidemiological studies. These considerations prompted us to examine the validity of information reported by a population of elderly persons with respect to the occurrence of cancer. Data from a cross-sectional epidemiological survey carried out in the French department of the Tarn (SW France) were cross-checked against data from a cancer registry of the same department used as reference.

### MATERIALS AND METHODS

A cross-sectional epidemiological survey was carried out in 1994 on persons 75 years of age and older, living either at home or in an institution in the French department of the Tarn (SW France). After stratification with respect to the (distribution of the) population of the canton (county) by residence, age and gender, 5161 persons were selected at random from the electoral registers of 41 communes (parishes) in the department. The subjects were informed by mail of the forthcoming visit of an investigator (physician or nurse) to their home, and the data were recorded on a questionnaire. Data from some individuals were recorded by telephone interview, and in certain cases, information was obtained from a near relative. Out of this population, 1129 persons could not be contacted owing to errors in electoral rolls from death ($n$=158), incorrect age ($n$=10), incorrect or change of address ($n$=542) or absence from home ($n$=419). Among those contacted, 664 did not respond owing to health problems ($n$=185) or other reasons ($n$=479). The overall participation was 65.3% (3368/5161).

*Received 5 February 1996*
*Revised 14 August 1996*
*Accepted 16 August 1996*

*Correspondence to:* F Berthier

Information about cancer was recorded from responses to the following question: 'Have you had a polyp, tumour, cyst, nodule or cancer?' and if 'yes', specify which: polyp, tumour, cyst, nodule or cancer, year of diagnosis and anatomical localization of the lesion(s), coded according to the International Classification of Diseases (ICD 9th revision). The 19 persons for whom the response to the last question was incomplete were put into the non-respondent group. The survey region was covered by a cancer registry set up in 1982, which recorded all cases of cancer apart from basal cell skin carcinoma.

The data provided by the 3349 respondents were compared with those in the cancer registry. The family and first names and dates of birth were cross-checked between the electoral registers and the cancer registry entries. Subjects were regarded as identified in the registry database if all three entries were in accord. To take errors in spelling of names into account, some subjects were considered to be included in the registry database despite lack of concordance of first name or date of birth, providing there was no discrepancy in place of birth. For each of the subjects identified in the registry database, the localization of the cancer and the year of diagnosis were recorded.

Using the registry data as reference, we examined the validity of the response that the person had, or had had, a cancer in terms of sensitivity and specificity. The influence of sex and anatomical localization on the declaration of cancer by the persons in the registry databases were analysed; the 95% confidence intervals and chi-square test were calculated using BMDP software (BMDP, Los Angeles, CA, USA).

### RESULTS

Details for the respondents and non-respondents are listed in Table 1. The results of the comparison between self-reports and registry data (Table 2) showed that only 78 of the 3349 respondents (2.3%) mentioned having a cancer since 1982, whereas 291 were included in the registry database (8.7%) since the same year. The prevalence of cancer from the self-reported data was underestimated by 73.6% relative to that calculated from the cancer registry data. The proportion of respondents identified on the registry database but

**Table 1** Details of respondents (R) and non-respondents (NR)

| | NR[a] (n=1644) | | | | | R (n=3349) | P[c] |
|---|---|---|---|---|---|---|---|
| | Incorrect or change of address and absence (n=961) | Refusal owing to health problems (n=185) | Refusal for other reasons (n=479) and response incomplete regarding cancer (n = 19) | P[b] | Total | | |
| Women (%) | 61.2 | 58.9 | 53.4 | < 0.05 | 58.6 | 52.6 | < 0.001 |
| Mean age | 83.3 | 84.2 | 81.4 | < 0.01 | 82.9 | 82.5 | < 0.05 |
| Subject identified in the registry database (%) | 7.5 | 9.2 | 6.0 | 0.33 | 7.2 | 8.7 | 0.21[d] |

[a]The deceased subjects (n=158) and age errors (n=10) were excluded. [b]Comparison among non-respondents. [c]Comparison between respondents and non-respondents. [d]According to sex.

**Table 2** Validity of the question 'Have you had a polyp, tumour, cyst, nodule or cancer?' – sole response 'cancer'

| Subject identified in the registry database | Self-report response 'cancer' | | | Sensitivity (%) | Specificity (%) |
|---|---|---|---|---|---|
| | Yes | No | Total | | |
| All cancers | | | | | |
| Yes | 60 | 231 | 291 | 20.6 (16.2–25.8)[a] | |
| No | 18 | 3040 | 3058 | | 99.4 (99.1–99.6)[a] |
| Cancer site (ICD-9) | | | | | |
| Urinary tract (188–189) | | | | | |
| Yes | 3 | 38 | 41 | 7.3 | |
| No | 0 | 3308 | 3308 | | 100.0 |
| Digestive tract (150–159) | | | | | |
| Yes | 10 | 52 | 62 | 16.1 | |
| No | 2 | 3285 | 3287 | | 99.9 |
| Haematological (200–208) | | | | | |
| Yes | 2 | 12 | 14 | 14.3 | |
| No | 3 | 3332 | 3335 | | 99.9 |
| Nose, ear, throat (140–149, 160–161) | | | | | |
| Yes | 6 | 16 | 22 | 27.3 | |
| No | 5 | 3322 | 3327 | | 99.8 |
| Breast and gynaecological (174, 179–184) | | | | | |
| Yes | 18 | 18 | 36 | 50.0 | |
| No | 0 | 1724 | 1724 | | 100.0 |
| Prostate (185) | | | | | |
| Yes | 15 | 82 | 97 | 15.5 | |
| No | 3 | 1489 | 1492 | | 99.8 |

[a]Numbers in brackets, are 95% confidence intervals.

not reporting cancer was 79.4%, producing a sensitivity of 20.6%. Among the 78 respondents mentioning the occurrence of cancer, 60 were identified in the cancer registry, giving a positive predictive value of 76.9%. Eighteen out of the 3058 persons not identified on the cancer registry reported the occurrence of cancer, giving a specificity of 99.4%.

Sensitivity was better for women than men (28.6% *vs.* 17.0%, $P < 0.05$). This difference was attributed to differences in the anatomical localization of the cancer (Table 2). Breast and gynaecological cancers tended to be well reported (sensitivity = 50%), and prostatic cancer was poorly reported (sensitivity = 15.5%), whereas there was no significant difference between the sexes for ear, nose and throat, haematological, digestive or urinary tract cancers (sensitivity = 14.9% for men *vs.* 15.4% for women, $P$ = 0.86). There was a high specificity (99.8–100%), irrespective of localization.

### DISCUSSION

Our study indicated that there were marked differences between the information on cancer reported by the elderly and that recorded in the cancer registry. The prevalence of cancer from self-reports

© Cancer Research Campaign 1997

of our elderly population (2.3%) was considerably below that determined from the registry (8.7%). Underestimates of self-reported prevalence have been reported and ranged from 13% (Paganini-Hill and Chao, 1993) to 47% (Kehoe et al, 1994), *vs* 74% in the present study. Indeed, only a fifth of patients with cancer in our population mentioned the occurrence of the condition (sensitivity = 20.6%), whereas this proportion ranged from 55% (Schrijvers et al, 1994) to 83% in other published studies (Paganini-Hill and Chao, 1993).

The first explanation for these differences (concerns) the advanced age of our population, since the studies mentioned above were all carried out on mixed age adult populations. Age is a source of inaccuracy owing to the higher frequency of cognitive and memory disorders in persons over 75 years of age, the reduced inclination to be informed about their disease (Cassileth et al, 1980) and more taboos surrounding cancer in this age group than in younger persons (McKenna, 1994). In addition, diagnostic and therapeutic problems in this age group tend to hinder transmission of an accurate diagnosis to the patients. Schrijvers et al (1994) showed that the underestimate of the prevalence of cancer was greatest in the highest age range in a study of persons aged between 15 and 74 years. In France, the true diagnosis is less commonly communicated to the patient than in other countries, which could also account for the differences (Holland et al, 1987).

The low sensitivities observed may also have been caused by a possible lack of completeness in the cancer registry. Out of the 18 false-positive subjects, only one did not reside in the Tarn department at the time of the diagnosis and was thus not included in the cancer registry. The status of the other 17 subjects could not be checked owing to legal difficulties, but even assuming that all the false positives did in fact have cancer, the sensitivity only rose slightly to 25.2% (78/309). The bias introduced by this possible lack of completeness was, therefore, assumed to be modest. Furthermore, the status of all respondents could readily be checked against the registry data. Using physicians' notes as reference may be less straight forward. For example, Kehoe et al (1994) found that almost a third of the reports could not be checked against physicians' records, as either the patients did not mention having a family physician or the physicians could not be contacted or refused to participate in the study.

Breast and gynaecological cancers were most frequently mentioned as being cancer. As reported by other authors (Colditz et al, 1986; Paganini-Hill and Chao, 1993; Schrijvers et al, 1994), the clear-cut diagnostic features of breast cancer may account for the fact that it is more accurately reported than other cancers.

Our results show the lack of precision of self-reported data on the prevalence of cancer in a very elderly population in France. The fact that cancer is a well-defined, serious condition is no guarantee of the validity of self-reports, since the term cancer is still taboo in France for both elderly patients and their physicians.

### ACKNOWLEDGEMENTS

This study was funded by the Fédération Nationale des Centres de Lutte contre le Cancer, the Caisse Centrale de Mutualité Agricole, the Caisse Maladie Régionale des Professions indépendantes and Pierre Fabre Laboratories. We would like to thank the Nursing Schools of Albi and Castres and the Union Mutualiste Tarnaise for their cooperation.

### REFERENCES

Cassileth BR, Zupkis RV, Sutton-Smith K and March V (1980) Information and participation preferences among cancer patients. *Ann Intern Med* **92**: 832–836

Colditz GA, Martin P, Stampfer MJ, Willett WC, Sampson L, Rosner B, Hennekens CH and Speizer FE (1986) Validation of questionnaire information on risk factors and disease outcomes in a prospective cohort study of women. *Am J Epidemiol* **123**: 894–900

Coleman MP and Lutz J-M (1996) Tendances évolutives du cancer et prévision des besoins: vers une meilleure utilisation des registres du cancer. *Rev Epidemiol Sante Pub* **44**: S2–S6

Holland JC, Geary N, Marchini A and Tross S (1987) An international survey of physician attitudes and practice in regard to revealing the diagnosis of cancer. *Cancer Invest* **5**: 151–154

Kehoe R, Wu S-Y, Leske MC and Chylack LT (1994) Comparing self-reported and physician-reported medical history. *Am J Epidemiol* **139**: 813–818

McKenna RJ (1994) Clinical aspects of cancer in the elderly. *Cancer* **74**: 2107–2117

Paganini-Hill A and Chao A (1993) Accuracy of recall of hip fracture, heart attack, and cancer: a comparison of postal survey data and medical records. *Am J Epidemiol* **138**: 101–106

Schrijvers CTM, Stronks K, Van De Mheen DH, Coebergh J-WW and Mackenbach JP (1994) Validation of cancer prevalence data from a postal survey by comparison with cancer registry records. *Am J Epidemiol* **139**: 408–414

Yancik R and Ries LA (1994) Cancer in older persons. Magnitude of the problem – How do we apply what we know? *Cancer* **74**: 1995–2003

© Cancer Research Campaign 1997


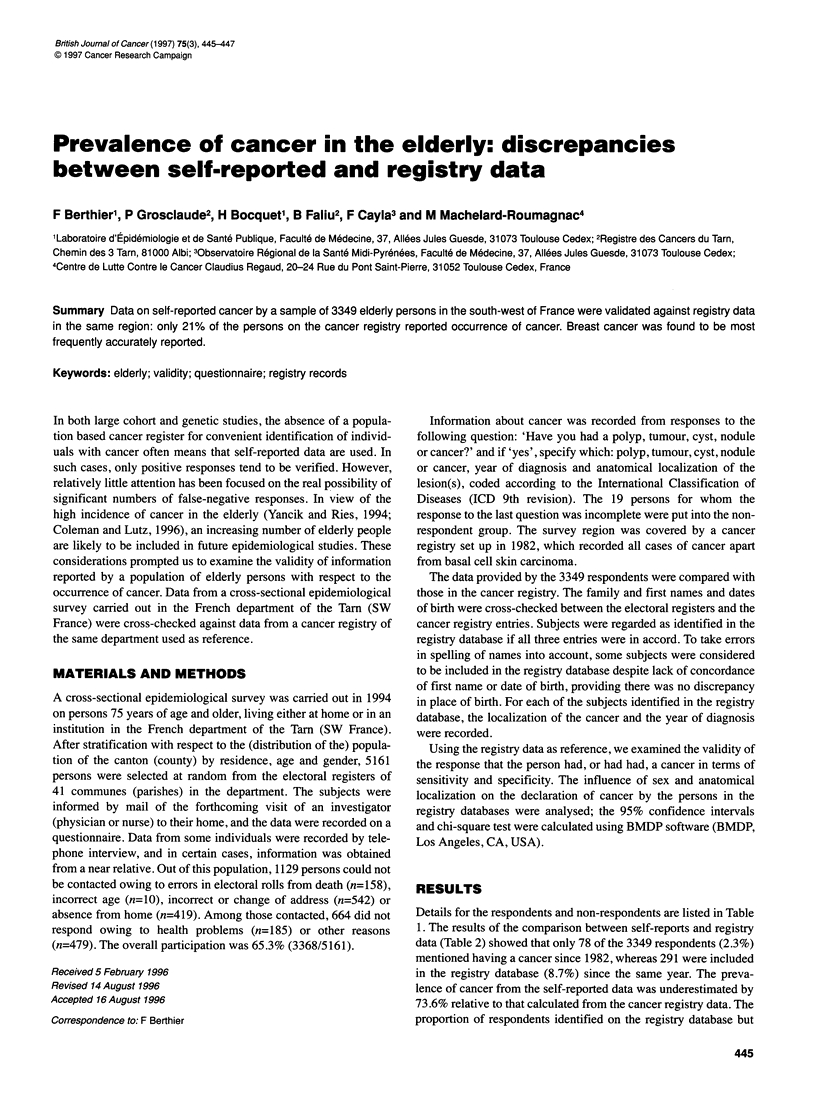

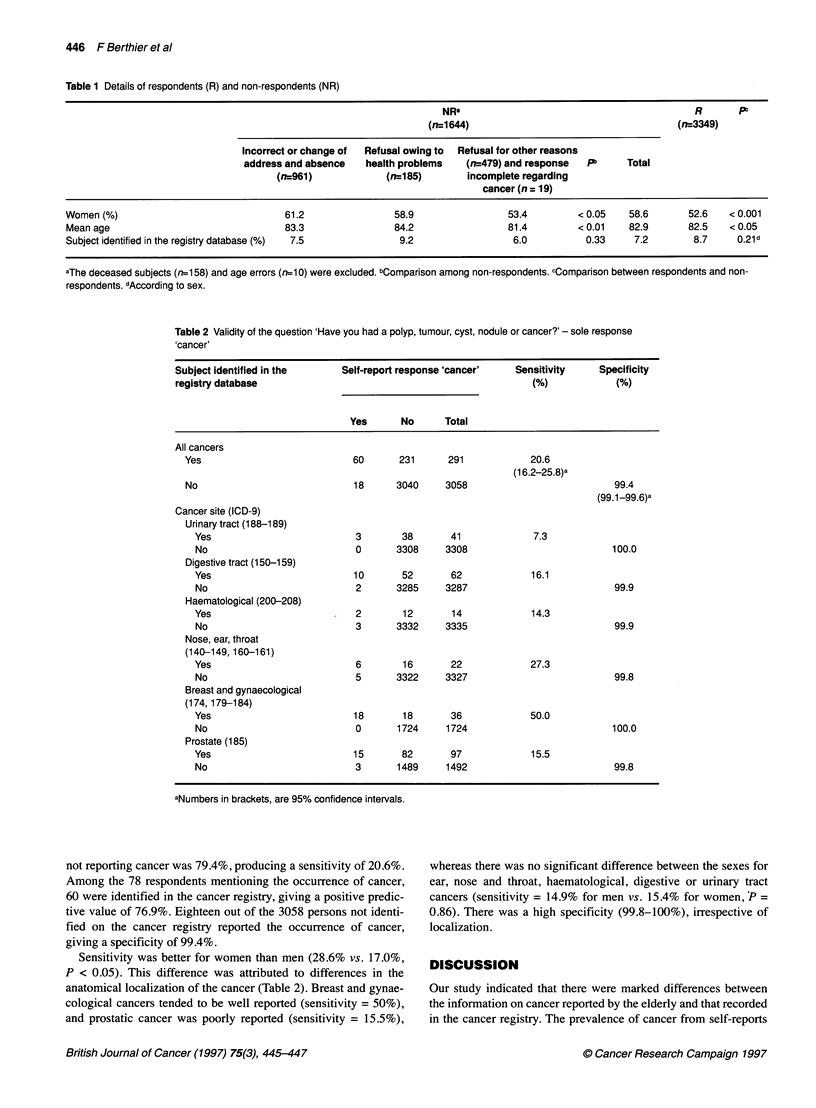

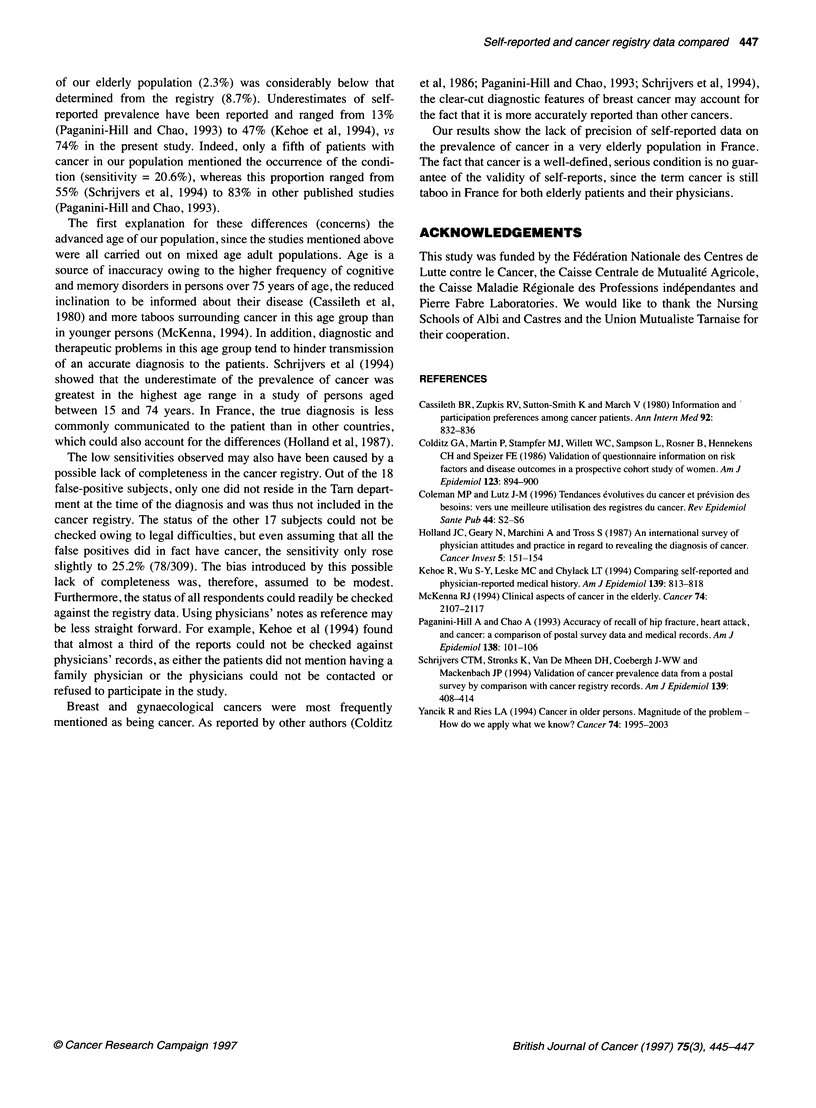


## References

[OCR_00280] Cassileth B. R., Zupkis R. V., Sutton-Smith K., March V. (1980). Information and participation preferences among cancer patients.. Ann Intern Med.

[OCR_00285] Colditz G. A., Martin P., Stampfer M. J., Willett W. C., Sampson L., Rosner B., Hennekens C. H., Speizer F. E. (1986). Validation of questionnaire information on risk factors and disease outcomes in a prospective cohort study of women.. Am J Epidemiol.

[OCR_00292] Coleman M. P., Lutz J. M. (1996). Tendances évolutives du cancer et prévision des besoins: vers une meilleure utilisation des registres du cancer.. Rev Epidemiol Sante Publique.

[OCR_00297] Holland J. C., Geary N., Marchini A., Tross S. (1987). An international survey of physician attitudes and practice in regard to revealing the diagnosis of cancer.. Cancer Invest.

[OCR_00302] Kehoe R., Wu S. Y., Leske M. C., Chylack L. T. (1994). Comparing self-reported and physician-reported medical history.. Am J Epidemiol.

[OCR_00305] McKenna R. J. (1994). Clinical aspects of cancer in the elderly. Treatment decisions, treatment choices, and follow-up.. Cancer.

[OCR_00309] Paganini-Hill A., Chao A. (1993). Accuracy of recall of hip fracture, heart attack, and cancer: a comparison of postal survey data and medical records.. Am J Epidemiol.

[OCR_00314] Schrijvers C. T., Stronks K., van de Mheen D. H., Coebergh J. W., Mackenbach J. P. (1994). Validation of cancer prevalence data from a postal survey by comparison with cancer registry records.. Am J Epidemiol.

[OCR_00320] Yancik R., Ries L. A. (1994). Cancer in older persons. Magnitude of the problem--how do we apply what we know?. Cancer.

